# Hysterectomy in women with disabilities: a systematic review

**DOI:** 10.1093/epirev/mxaf020

**Published:** 2026-01-06

**Authors:** Jayati Khattar, Carmela Melina Albanese, Kathryn Barrett, Natalie V Scime, Hilary K Brown

**Affiliations:** Dalla Lana School of Public Health, University of Toronto, Toronto, Ontario, Canada; Dalla Lana School of Public Health, University of Toronto, Toronto, Ontario, Canada; Library, University of Toronto Scarborough, Toronto, Ontario, Canada; Department of Obstetrics & Gynecology, Cumming School of Medicine, University of Calgary, Calgary, Alberta, Canada; Dalla Lana School of Public Health, University of Toronto, Toronto, Ontario, Canada; Department of Health & Society, University of Toronto Scarborough, Toronto, Ontario, Canada

**Keywords:** persons with disability, hysterectomy, women’s health, health equity

## Abstract

Hysterectomy is the most frequently performed nonobstetric surgery in women. Women with disabilities face barriers to reproductive health care, and little is known about their hysterectomy risk. The objective of this systematic review was to compare hysterectomy risk among women with and without disabilities. We searched the MEDLINE, Embase, PsycInfo, and CINAHL Plus databases from inception to May 2024 using validated search strategies. We included peer-reviewed observational studies that compared hysterectomy in women with physical, sensory, cognitive, and intellectual or developmental disabilities with those without disabilities. Study characteristics and data were extracted using a standardized form; the Newcastle-Ottawa Scale (NOS) was used for quality assessment. Study findings were summarized narratively following Synthesis Without Meta-analysis guidelines. The search yielded 3686 unique records, of which 5 met our criteria. These included 1 retrospective cohort and 4 cross-sectional studies, which were conducted in the United States (*n* = 3), Canada (*n* = 1), and South Korea (*n* = 1), and ranged in size from 881 to 42 842 participants. Evidence from 4 studies indicated hysterectomy frequency was higher among women with disabilities (range: 6.1% to 22.8%) compared with those without disabilities (range: 2.2% to 18.6%). Three studies suggested the disparity in hysterectomy was greatest among premenopausal women. Quality assessment scores on the NOS ranged from 0 to 8 (median, 3), with limitations mostly related to measurement of the exposure and outcome. The limited research on this topic points to the need for more studies on hysterectomy among women with disabilities, given historical reproductive injustices faced by this population.

## Introduction

Hysterectomy is the most frequently performed nonobstetric surgery in women.^1-4^ The surgery is typically undertaken for benign indications (90%), most often uterine fibroids, abnormal bleeding, and endometriosis.^5-7^ Although rates of hysterectomy have declined in recent years with the rise of nonsurgical methods to manage these conditions, 45% of women will have a hysterectomy in their lifetime.^7-11^ Hysterectomy carries imminent risks of postoperative complications such as infection^12^ and long-term risks of cardiometabolic disease,^13^^,^^14^ osteoporosis,^15-17^ and dementia,^18^^,^^19^ particularly when performed in premenopausal women.^20^

Previous studies have noted considerable differences in hysterectomy rates according to various sociodemographic characteristics, including education, race/ethnicity, health insurance, and geography.^21-23^ However, few studies have characterized hysterectomy rates among women with disabilities. Disability is defined by the World Health Organization as a complex interaction between health conditions and the environment, resulting in activity limitations or participation restrictions.^24^ The research gap regarding disability and hysterectomy is problematic because studies have found that women with disabilities face numerous disparities in reproductive health care access, with lower rates of cervical and breast cancer screening, use of a narrower range of contraceptive methods, and higher rates of sterilization procedures compared with their peers without disabilities.^25-29^ In qualitative studies, women with disabilities have reported lack of health care provider training and stigma associated with disability and sexuality as contributing to these disparities.^30^ Notably, women with disabilities also have elevated rates of menstrual disorders that are known indications for benign hysterectomy.^31-33^ These factors may influence provider or patient decisions related to hysterectomy for women with disabilities, but how their rates of hysterectomy compare with those without disabilities is underexplored.

Therefore, to address this critical research gap, we conducted a systematic review to compare hysterectomy rates among women with disabilities, including physical, sensory, cognitive, intellectual, or developmental disabilities, relative to those without disabilities. Such data are critical for understanding reproductive health disparities experienced by women with disabilities, to inform tailored and inclusive care.^34^

## Methods

### Information sources and search strategy

The review protocol was registered in the International Prospective Register of Systematic Reviews (PROSPERO) database (CRD42024545233). The search strategy was constructed in consultation with an expert research librarian and included subject headings and keywords related to disabilities, such as “physical disability/,” “sensory impair*,” and “cognitive impair*,” based on a search strategy that had been previously validated,^35^ as well as subject headings and keywords related to hysterectomy (Tables S1-S4). We searched the Ovid MEDLINE, Ovid Embase, Ovid APA PsycInfo, and EBSCO CINAHL Plus databases from inception to May 3, 2024. The reference lists of studies included after screening were also hand searched. This review follows the Synthesis Without Meta-Analysis guidelines.^36^ Covidence software was used to identify and remove duplicates.^37^

### Eligibility criteria and screening

Title, abstract, and full-text screening were completed independently by 2 authors (J.K. and C.M.A.), with conflicts resolved by a third author (H.K.B.). To be included, studies had to meet the following criteria: (1) include women with disabilities aged 12 years or older; (2) have a comparison group of women without disabilities; (3) include hysterectomy as an outcome; (4) be a peer-reviewed observational study (ie, cohort, cross-sectional, or case-control study); and (5) be written in English or French. Disability was defined as the presence of any physical, sensory, cognitive, intellectual, and/or developmental disability. Studies could focus on any hysterectomy type (eg, total, subtotal, radical), method (eg, abdominal, laparoscopic), or clinical indication (ie, benign [eg, abnormal uterine bleeding, chronic pelvic pain, endometriosis, uterine fibroids, uterine prolapse] or cancerous [eg, endometrial cancer, cervical cancer]). Studies could also report on any concomitant procedures (eg, bilateral salpingo-oophorectomy, tubal ligation).

### Data extraction

Data extraction was completed by 2 authors (J.K. and C.M.A.) independently using a standardized form created a priori, with conflicts resolved by a third author (H.K.B.). The form included the following study characteristics: authors and publication date, study region, study period, design, data sources, inclusion and exclusion criteria, number of participants, follow-up rates (if applicable), approach to missing data, measurement of disability, measurement of hysterectomy (including any details related to type, surgical method, clinical indication, or concomitant procedures examined), and confounders. Study findings we extracted were percentage of hysterectomy (overall and by subtype) in women with and without disabilities, and unadjusted and adjusted measures of association between disability status and the outcomes.

### Quality assessment

The Newcastle-Ottawa Scale (NOS) was completed independently by 2 authors (J.K. and C.M.A.), with conflicts resolved by a third author (H.K.B.).^38^ For cohort studies, the maximum possible score on the NOS is 9 (Table S5), with 4 stars allocated for selection and representativeness of the cohort, 3 for measurement of the outcome, and 2 for comparability of the exposed and unexposed groups. In our review, the first star for comparability of the groups was awarded based on adjustment for age, with a second star awarded if the study considered comorbid conditions or socioeconomic status as additional confounders. Similar to prior research,^39^^,^^40^ the NOS was amended for use with cross-sectional studies, with a maximum possible score of 5 stars (Table S5).

### Data analysis

Because of the small number of studies with heterogeneous designs, meta-analysis could not be performed. Therefore, we undertook a narrative synthesis following the Synthesis Without Meta-Analysis guidelines.^36^

## Results 

### Study selection

**Figure 1:**
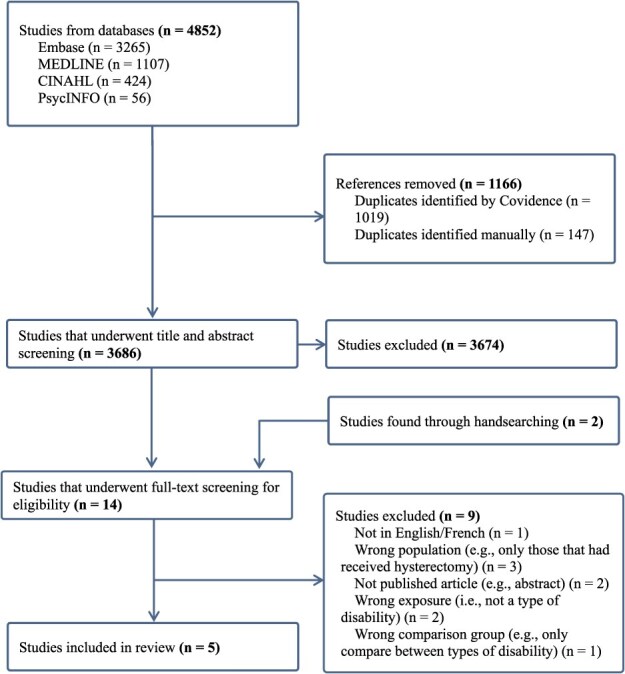
Preferred Reporting Items for Systematic Reviews and Meta-Analyses flow diagram detailing the study selection process.

The initial database search yielded 4852 records. After removing 1116 duplicates, 3686 unique records remained. After title and abstract screening, 12 articles were included in full-text screening. Two additional articles were found through hand-searching. After completing full-text screening, 5 articles were ultimately included in the review after excluding 9 for having the wrong population (*n* = 3) or exposure (*n* = 2), not having a comparison group (*n* = 1), not being a peer-reviewed study (*n* = 2), or not being written in English or French (*n* = 1) (Table S6 and Figure 1).^10^^,^^41-48^

### Study characteristics

The characteristics of the 5 included studies are summarized in Table 1.^49-53^ Three of the studies were conducted in the United States,^50-52^ 1 in Canada,^53^ and 1 in South Korea.^49^ Four of the studies were cross-sectional in design,^50-53^ and 1 was a retrospective cohort study.^49^ The cross-sectional studies used surveys (the National Survey of Family Growth, National Study of Women with Physical Disabilities, National Health Interview Survey, and Canadian Community Health Survey),^50-53^ and the retrospective cohort study used health administrative data from the Korean National Health Insurance Service.^49^ The inclusion criteria of most studies were similar, focusing broadly on all women with disabilities who were aged 15 to 65 years. However, Choi et al. restricted their cohort to women diagnosed with cervical cancer,^49^ and Nosek et al. provided little information on their inclusion criteria.^51^ Sample sizes ranged from 881 to 42 842 participants. The studies of survey data used self-reported questions about activity limitations or participation restrictions to measure disability status.^50^^,^^52^^,^^53^ In contrast, Choi et al. ascertained disability status using registration with a national welfare benefits program.^49^ All studies except for that of Choi et al. used self-reported data to ascertain hysterectomy status.^49^ No studies provided data on the type or surgical method of hysterectomy. Likewise, information on clinical indication was only provided by Choi et al., who focused on hysterectomy as part of cervical cancer treatment.^49^ Little information was available from the studies about the use of concomitant procedures, with the exception of Li et al., who reported hysterectomy alone and hysterectomy combined with tubal ligation.^50^

### Quality assessment

The results of the quality assessment process are summarized in Table 2. The scores of the cross-sectional studies ranged from 0 to 3 (median score, 3); the retrospective cohort study had a score of 8. Most studies (*n* = 4) used data representative of the general population.^49^^,^^50^^,^^52^^,^^53^ With respect to study-group comparability, most studies (*n* = 4) accounted for confounding by age by either adjustment in multivariable models or matching, and also adjusted for confounders such as income, education, and comorbid conditions.^49^^,^^50^^,^^52^^,^^53^ However, the studies largely used self-reported data to measure disability and hysterectomy, with few details on the validity of their approaches.^50^^,^^52^^,^^53^ They also did not provide information about nonrespondents or missing data; they largely used complete-case analysis.^49-52^

**Table 1:** Summary of the characteristics of included studies (*n* = 5).

**Study (First Author, Year)**	**Region**	**Study period, design, and data source**	**Inclusion criteria**	**Exclusion criteria**	**Analytic sample size (no.) and approach to missing data**	**Exposure (disability) measurement**	**Outcome (hysterectomy) measurement and characteristics**	**Confounders**
Choi et al., 2021^49^	South Korea	Retrospective cohort, Korean National Health Insurance Service, Korea National Disability Registration System, and Central Cancer Registry 2009-2013	Women aged ≥19 years and diagnosed with cervical cancer	Diagnosed with cervical cancer before 2005, aged <19 years at diagnosis or index date, prior history of other cancers (excluding thyroid cancer), heart or lung disabilities, missing data	16 767 Complete case analysis	Registration in the Korea National Disability Registration System	Health care administrative data Type: N/R Method: N/R Indication: cervical cancer	Age (matched design)
Li et al., 2018^50^	United States	Cross-sectional, National Survey of Family Growth 2011-2015	Women aged 15 to 44 years	Currently or trying to become pregnant, medically or surgically sterile for noncontraceptive reasons, had male partners who were medically or surgically sterile, missing data	9971 Complete case analysis	Self-report, survey questions	Self-report, survey question Type: N/R Method: N/R Indication: N/R	Age, race and ethnicity, education, health insurance status, poverty status, marital status, parity, and self-rated health status
Nosek et al., 2001^51^	United States	Cross-sectional, National Study of Women with Physical Disabilities 1992-1996	Women aged 18 to 65 years, physical disabilities related to mobility	Disability not mobility related	881 Complete case analysis	Self-report	Self-report, survey question Type: N/R Method: N/R Indication: N/R	None
Rivera Drew et al., 2013^52^	United States	Cross-sectional, National Health Interview Series 2000, 2005, 2010	Women aged ≥18 years	Women with diagnosis of any condition related to uterus, fallopian tubes, or ovaries, already had a hysterectomy, missing data	42 842 Complete case analysis	Self-report, survey questions	Self-report, survey question Type: N/R Method: N/R Indication: N/R	Education, incidence and timing of first birth, race or ethnicity, and survey wave
Scime et al., 2021^53^	Canada	Cross-sectional, Canadian Community Health Survey 2012	Women aged ≥20 years	Missing data	12 813 Multiple imputation	Self-report, survey questions	Self-report, survey question Type: N/R Method: N/R Indication: N/R	Income, education, minority status, marital status, employment, age-by-disability interaction term

Abbreviation: N/R, not reported.

**Table 2:** Quality assessment using the Newcastle-Ottawa Scale for the included studies (*n* = 5).

**Study (First Author, Year)**	**1) Representativeness of the exposed cohort/participants**	**2) Selection of the nonexposed cohort**	**3) Ascertainment of exposure (disability)**	**4) Demonstration that outcome of interest was not present at start of study**	**5) Comparability of cohorts on the basis of the design or analysis**	**6) Assessment of outcome (hysterectomy)**	**7) Follow-up long enough for outcomes to occur**	**8) Adequacy of follow-up of cohorts**	**Final score**
Choi et al., 2021^49^	*	*	*	*	*	*	*	*	8
Li et al., 2018^50^	*	N/A		N/A	**		N/A	N/A	3
Nosek et al., 2001^51^		N/A		N/A			N/A	N/A	0
Rivera Drew et al., 2013^52^	*	N/A		N/A	**		N/A	N/A	3
Scime et al., 2021^53^	*	N/A		N/A	**		N/A	N/A	3

Abbreviation: N/A, not applicable.

### Synthesis of findings

The results of the included studies are summarized in Table 3. Four studies suggested women with disabilities had an elevated likelihood of having a hysterectomy. These studies reported that the frequency of hysterectomy in women with disabilities ranged from 6.1% to 22.8%, whereas for women without disabilities, it ranged from 2.2% to 18.6%. Although the measures of association varied, adjusted estimates suggested women with disabilities were 1.12 to 2.18 times more likely to have a hysterectomy compared with those without disabilities. In the study by Choi et al., in which they specifically examined hysterectomy following a diagnosis of cervical cancer, the unadjusted odds of hysterectomy were 0.82 comparing women with disabilities with those without disabilities.^49^

**Table 3:** Percentage of women with hysterectomy by disability status and the respective measures of association, with selected sub-group analyses.

**Study (First Author, Year)**	**Exposure groups**	**With hysterectomy, %**	**Measure of association**	**Unadjusted (95% CI)**	**Adjusted (95% CI)**
Choi et al., 2021^49^	Disability No disability	41.4 46.3	Odds ratio	0.82^*^ Referent	N/R N/R
Li et al., 2018^50^	Disability No disability	6.1 2.2	Odds ratio	2.87^*^ Referent	N/R N/R
Nosek et al., 2001^51^	Disability No disability	22.0 12.0	Prevalence ratio	1.83^*^ Referent	N/R N/R
Rivera Drew et al., 2013^52^	Disability No disability	11.5 18.6	Hazard ratio	N/R N/R	1.14 (1.06-1.22) Referent
Scime et al., 2021^53^	Disability No disability	22.8 11.1	Prevalence ratio	2.05^*^ Referent	N/R N/R
**Sub-group analyses (selected)**					
Li et al., 2018^50^	Outcome: Hysterectomy Noncognitive disabilities Cognitive disabilities No disability	6.2 6.0 2.2	Odds ratio	2.96^*^ 2.83^*^ Referent	1.65 (0.96-2.84) 2.64 (1.53-4.56) Referent
Li et al., 2018^50^	Outcome: Hysterectomy and tubal ligation Disability Noncognitive disabilities Cognitive disabilities No disability	4.7 5.3 4.5 1.4	Odds ratio	3.54^*^ 3.95^*^ 3.34^*^ Referent	N/R N/R N/R N/R
Rivera Drew et al., 2013^52^	Types of disability: Sensory/cognitive/developmental/mental Musculoskeletal Arthritis-related Other Multiple No disability	N/R N/R N/R N/R N/R 18.6	Hazard ratio	N/R N/R N/R N/R N/R Referent	0.76 (0.55-1.06) 1.01 (0.87-1.18) 1.05 (0.89-1.24) 0.95 (0.79-1.13) 1.30 (1.20-1.42) Referent
Scime et al., 2021^53^	Age stratified (years): Disability (20-44) No disability (20-44) Disability (45-59) No disability (45-59) Disability (≥60) No disability (≥60)	4.0 1.7 20.2 13.2 38.6 32.5	Prevalence ratio	2.32 (1.47-3.67) Referent 1.52 (1.27-1.83) Referent 1.19 (1.09-1.30) Referent	2.18 (1.36-3.50) Referent 1.48 (1.21-1.80) Referent 1.12 (1.02-1.24) Referent

^*^These estimates were calculated by the review’s authors.

Three studies found the strength of the association between disability and hysterectomy diminished with age. In the study by Scime et al., the prevalence of hysterectomy was 2.18 (95% CI, 1.36-3.50) times higher among women with disabilities aged 20 to 44 years, relative to their same-aged counterparts.^53^ However, among women aged 60 years or older, the prevalence ratio decreased to 1.12 (95% CI, 1.02, 1.24).^53^ Rivera Drew et al. found the hazard ratios for the association between disability and hysterectomy decreased from 2.43 (95% CI, 1.63-3.64) at age 21 to 25 years to 1.30 (95% CI, 1.05-1.60) at 41 to 45 years, and most women with disabilities who were 46 years of age or older did not have an elevated risk of hysterectomy.^52^ Although Nosek et al. did not report age-stratified results, they indicated in their article that women with and those without disabilities who were aged 35 years or older had a similar risk of hysterectomy.^51^ Finally, although Li et al. did not stratify their results by age, they reported the average age at hysterectomy was approximately 2 years younger in women with disabilities compared with those without disabilities.^50^

Type and/or severity of disability was considered by 3 studies. Li et al. found higher odds of hysterectomy among women with cognitive, but not other, disabilities.^50^ Rivera Drew et al. reported elevated risk of hysterectomy among women with multiple disabilities, but not other individual disabilities.^52^ Finally, Scime et al. found little variation in the effect estimates by disability type (functional or activity limiting disability) or severity levels.^53^

## Discussion

### Summary of findings

The findings of the present systematic review of 5 studies from the United States, Canada, and South Korea suggest women with disabilities, and particularly premenopausal women, have elevated risk of hysterectomy compared with women without disabilities. There was limited information on the association by disability type or severity, or by type or surgical method of hysterectomy, and the quality of evidence was low. Only 1 study examined hysterectomy in the context of a specific clinical indication.^49^ Given the frequency of hysterectomy and its important implications for women’s health, our review highlights the need for more high-quality research in this area.

### Comparison with previous literature

To our knowledge, this is the first systematic review on disability and hysterectomy. Similar to the studies included in our review, a non–peer-reviewed report using data from the 2021 National Health Interview Survey found the lifetime prevalence of hysterectomy was 20.9% in women with disabilities compared with 14.1% in women without disabilities.^10^ Research on complications of hysterectomy among women with disabilities also suggests women with disabilities are more likely to undergo open hysterectomies compared with less invasive methods.^41^ Our review’s findings also align with studies that have repeatedly found evidence of reproductive health disparities among women with disabilities, including lower rates of cervical and breast cancer screening,^26^^,^^29^ elevated rates of sexual abuse,^54^ use of a narrower range of and more invasive contraceptive methods,^25^^,^^27^^,^^28^ and lower utilization of prenatal care services.^55^ Access to education and care is a major contributor to these disparities: young people with disabilities report receiving little sexual health education in school.^56^ Women with disabilities are also less likely to access reproductive health care in primary care settings: they report lack of transportation, inaccessible health care spaces, and insensitivity from health care providers as obstacles.^57^

### Reasons for findings

Multiple factors may contribute to the elevated likelihood of hysterectomy among women with disabilities. Women with disabilities may be more likely than those without disabilities to have certain benign clinical indications for the procedure, such as irregular menstrual bleeding, dysmenorrhea, and endometriosis,^33^^,^^58-60^ which account for 90% of hysterectomies performed.^6^^,^^7^ Shared etiologies between certain disabilities and gynecologic disorders (eg, autoimmune disorders and endometriosis) may contribute to the elevated prevalence of these clinical indications.^61^^,^^62^ Benign hysterectomy, therefore, may be indicated more frequently in women with disabilities than in their peers without disabilities. Fewer studies have examined cancerous indications for hysterectomy in women with disabilities, such as cervical, endometrial, or ovarian cancer.^63^ Choi et al. found that fewer women with, versus without, disabilities who were diagnosed with cervical cancer underwent the procedure, suggesting a gap in access to cancer treatment.^49^ However, more broadly, limited information on the indication for hysterectomy across the included studies makes it difficult to draw conclusions about the decisions or mechanisms that led to the observed disparities.

Women with disabilities may also be more likely to undergo hysterectomy for reasons that are not medically indicated, leading to concerns about their reproductive autonomy. For example, although hysterectomy as a contraceptive method is not recommended, women with disabilities have reported receiving a hysterectomy for the purpose of birth control,^64-66^ citing social pressure to avoid pregnancy.^51^ Likewise, women with some disabilities may undergo hysterectomy due to patient or caregiver concerns about menstrual hygiene or behavioral fluctuations associated with menstruation.^31^^,^^58^^,^^67^ Caregiver concern about sexual abuse and unintended pregnancy may also be a contributing factor.^31^^,^^64^^,^^68^ Providers report that use of oral contraception to manage such concerns is complicated by adherence-related difficulties; insertion of intrauterine devices may require sedation; and use of progestin injections is associated with negative side effects.^61^^,^^69^^,^^70^ Such concerns may contribute to provider, caregiver, and/or patient decisions about hysterectomy. Further research is needed on this topic.

Critically, women with disabilities often face difficulties advocating for their reproductive health and alternative, less invasive methods of treatment. Communication barriers and time constraints during clinical encounters can limit effective discussion with health care providers.^30^ Furthermore, implicit or explicit biases may influence how health care providers offer reproductive health care services to women with disabilities.^71^^,^^72^ For example, women with disabilities have reported that providers express negative attitudes about disability and sexual and reproductive health.^73^ These attitudes can prevent women with disabilities from freely discussing their reproductive health concerns. Notably, obstetricians and gynecologists receive little training on disability.^74^^,^^75^ For example, in a 2016 survey of 322 obstetricians and gynecologists in the United States, only 17.2% reported receiving any disability-specific training.^74^ This lack of disability-related knowledge and implicit bias may lead to suboptimal treatment decisions. For example, the particularly large disparity in hysterectomy rates between women with and without disabilities at premenopausal ages may be partly explained by a lower provider threshold for surgical interventions with benign gynecologic indications,^47^^,^^65^^,^^76^ despite clinical guidelines that recommend the use of less invasive, fertility-preserving treatment options in this age group.^9^^,^^77^

### Implications

The higher frequency of hysterectomy among women with disabilities observed in this systematic review, and particularly among premenopausal women, is concerning. Gynecologic concerns at younger ages are largely related to menstrual disorders, which can often be managed through a trial of alternative methods, such as anti-inflammatory medications, hormonal therapies, and radiologic procedures.^78-80^ Moreover, the long-term risks associated with hysterectomy are more pronounced when the procedure is done at younger ages.^20^ According to the American College of Obstetrics and Gynecology, management of menstruation for women with disabilities should begin with the least invasive and reversible methods, in the same manner that it would for women without disabilities.^77^ If surgery is recommended, forms of surgery that do not affect fertility should be prioritized.^81^ If a hysterectomy is judged to be the most appropriate clinical option, the decision to proceed with the procedure must be patient centered and occur only with the patient’s informed consent.^82^ Clear and accessible communication with the patient is critical, because, despite legal protections, reproductive health decisions may still be highly influenced by caregivers.^83-85^ Although multiple factors may contribute to the elevated rates of hysterectomy among women with disabilities, the observed disparity is situated within a broader context of reproductive health disparities for women with disabilities that require attention. Addressing these disparities requires structural changes, including the provision of accessible sexual health education to people with disabilities^75^ and the expansion of health care provider curricula to include disability-related training and reproductive justice concepts.^20^^,^^73^

The limited number of studies identified in this review shows the need for more research in this area. Future studies should take a longitudinal approach, because cross-sectional data help researchers disentangle whether the disability proceeded the hysterectomy or was a consequence of it.^15^ Future studies should also examine type of hysterectomy and surgical approach, because minimally invasive methods are recommended,^86^ and previous studies have found disparities in the use of minimally invasive methods according to socioeconomic factors.^41^^,^^87^ Studies should also further examine the varying clinical indications (ie, both benign and cancerous) for hysterectomy in women with disabilities to understand the clinical scenarios and decision-making that may lead to disparities. Information on these indications will help shed light on whether disparities are explained by underlying health concerns, gaps in access to services, or potentially avoidable health care provider decisions. All this research must be disaggregated by disability type and severity, given the potential heterogeneity in risk factors and experiences among women with disabilities.

### Strengths and limitations

A key strength of this review is the use of validated search strategies^35^ to identify peer-reviewed studies to answer a novel research question and application of the NOS to evaluate the quality of evidence.^38^ However, the few studies identified and the heterogeneity of their methods meant meta-analysis was not feasible. The included studies used samples drawn from the general population and addressed important confounders, such as age and education. The studies were largely cross-sectional, which limits our insight into the temporal relationship between disability and hysterectomy. Moreover, little information was available about the type or surgical method of hysterectomy, as well as indications for the procedure. Lastly, the included cohorts were drawn from diverse populations, with varying health care systems, meaning the results are not specific to a particular context.

## Conclusion

This systematic review identified 5 studies that examined the association between disability and hysterectomy, with the results potentially suggesting women with disabilities may have a higher likelihood of having a hysterectomy, particularly at younger ages. The results must be cautiously interpreted, because the studies were limited in number and quality, and stronger evidence is needed to address this critical research gap and ultimately ensure equitable access to reproductive health care for women with disabilities.

## Supplementary Material

Web_Material_mxaf020

## Data Availability

Because this was a systematic review, no new data were generated or analyzed in support of this research.
