# Supplementary material for: Hysterectomy in women with disabilities: a systematic review
Source: Epidemiol Rev. 2026 Jan 6;48(1):mxaf020. doi: 10.1093/epirev/mxaf020 (PMC12858371; doi:10.1093/epirev/mxaf020)
Supplement: Web_Material_mxaf020 [file web_material_mxaf020.docx]

**Hysterectomy in women with disabilities: a systematic review**

**Authors**: Jayati Khattar, Carmela Melina Albanese, Kathryn Barrett, Natalie V. Scime, Hilary K. Brown

**Table of Contents**

**Table S1:** Search strategies in OVID MEDLINE

**Table S2:** Search strategies in OVID Embase

**Table S3:** Search strategies in OVID APA PyscINFO

**Table S4:** Search strategies in EBSCO CINAHL Plus

**Table S5**: Details of the Newcastle Ottawa Scale

**Table S6:** Studies that were excluded during full-text screening and reasons for exclusion

**References**

**Supplementary Material**

**Table S1: Search strategies in OVID Medline**

Ovid MEDLINE: Epub Ahead of Print, In-Process & Other Non-Indexed Citations, Ovid MEDLINE® Daily and Ovid MEDLINE® <1946-Present>

| **#** | **Query** | **Results from 2 May 2024** |
| --- | --- | --- |
| 1 | Disabled persons/ | 48,776 |
| 2 | "Activities of daily living"/ | 74,673 |
| 3 | disabled.tw,kf. | 27,390 |
| 4 | disabilit*.tw,kf. | 259,657 |
| 5 | handicap*.tw,kf. | 27,005 |
| 6 | (activit* adj1 daily living).tw,kf. | 2,044 |
| 7 | Mobility limitation/ | 5,340 |
| 8 | (physical* adj limit*).tw,kf. | 3,431 |
| 9 | (physical* adj impair*).tw,kf. | 3,280 |
| 10 | motor limitation*.tw,kf. | 106 |
| 11 | motor impair*.tw,kf. | 9,777 |
| 12 | mobility limit*.tw,kf. | 1,837 |
| 13 | mobility impair*.tw,kf. | 1,449 |
| 14 | functional limitation*.tw,kf. | 9,013 |
| 15 | (functional* adj impair*).tw,kf. | 25,074 |
| 16 | activity limitation*.tw,kf. | 4,083 |
| 17 | participation limitation*.tw,kf. | 101 |
| 18 | (walk* adj difficult*).tw,kf. | 706 |
| 19 | ambulation difficult*.tw,kf. | 41 |
| 20 | dependent ambulation/ | 207 |
| 21 | self-help devices/ or exp wheelchairs/ | 11,082 |
| 22 | dependent ambulation.tw,kf. | 27 |
| 23 | assistive technolog*.tw,kf. | 3,880 |
| 24 | assistive device*.tw,kf. | 3,712 |
| 25 | wheel chair*.tw,kf. | 391 |
| 26 | wheelchair*.tw,kf. | 9,086 |
| 27 | mobility aid*.tw,kf. | 513 |
| 28 | mobility equipment.tw,kf. | 39 |
| 29 | mobility technolog*.tw,kf. | 56 |
| 30 | self help device*.tw,kf. | 227 |
| 31 | (walk* adj aid*).tw,kf. | 1,422 |
| 32 | amputees/ | 4,409 |
| 33 | amput*.tw,kf. | 55,920 |
| 34 | paraplegia/ | 13,276 |
| 35 | quadriplegia/ | 8,457 |
| 36 | paraplegi*.tw,kf. | 19,695 |
| 37 | quadr#plegi*.tw,kf. | 4,757 |
| 38 | tetraplegi*.tw,kf. | 5,038 |
| 39 | persons with mental disabilities/ | 3,707 |
| 40 | exp intellectual disability/ | 107,303 |
| 41 | cognitive dysfunction/ | 39,958 |
| 42 | (mental* adj retard*).tw,kf. | 35,589 |
| 43 | (cognitive* adj impair*).tw,kf. | 101,276 |
| 44 | cognitive disorder*.tw,kf. | 6,693 |
| 45 | cognitive dysfunction*.tw,kf. | 23,051 |
| 46 | developmental disabilities/ | 22,659 |
| 47 | (development* adj disorder*).tw,kf. | 11,986 |
| 48 | developmental delay*.tw,kf. | 19,977 |
| 49 | learning disabilities/ | 14,739 |
| 50 | learning disorder*.tw,kf. | 1,697 |
| 51 | academic disorder*.tw,kf. | 3 |
| 52 | persons with hearing impairments/ | 3,140 |
| 53 | hearing loss/ | 20,788 |
| 54 | deafness/ | 29,568 |
| 55 | hearing impair*.tw,kf. | 16,809 |
| 56 | hearing loss.tw,kf. | 60,131 |
| 57 | deaf*.tw,kf. | 42,795 |
| 58 | hard of hearing.tw,kf. | 1,959 |
| 59 | visually impaired persons/ | 2,792 |
| 60 | vision disorders/ | 31,171 |
| 61 | blindness/ | 21,764 |
| 62 | vision, low/ | 4,166 |
| 63 | (visual* adj impair*).tw,kf. | 18,034 |
| 64 | vision impair*.tw,kf. | 3,461 |
| 65 | vision disorder*.tw,kf. | 818 |
| 66 | blind*.tw,kf. | 362,948 |
| 67 | vision loss.tw,kf. | 13,378 |
| 68 | low vision.tw,kf. | 3,615 |
| 69 | deaf-blind disorders/ | 175 |
| 70 | deafblind*.tw,kf. | 157 |
| 71 | deaf blind*.tw,kf. | 399 |
| 72 | sensory loss*.tw,kf. | 3,733 |
| 73 | sensory impair*.tw,kf. | 2,812 |
| 74 | 1 or 2 or 3 or 4 or 5 or 6 or 7 or 8 or 9 or 10 or 11 or 12 or 13 or 14 or 15 or 16 or 17 or 18 or 19 or 20 or 21 or 22 or 23 or 24 or 25 or 26 or 27 or 28 or 29 or 30 or 31 or 32 or 33 or 34 or 35 or 36 or 37 or 38 or 39 or 40 or 41 or 42 or 43 or 44 or 45 or 46 or 47 or 48 or 49 or 50 or 51 or 52 or 53 or 54 or 55 or 56 or 57 or 58 or 59 or 60 or 61 or 62 or 63 or 64 or 65 or 66 or 67 or 68 or 69 or 70 or 71 or 72 or 73 | 1,270,817 |
| 75 | exp hysterectomy/ | 34,327 |
| 76 | hysterectom*.tw,kf. | 44,507 |
| 77 | 75 or 76 | 56,139 |
| 78 | 74 and 77 | 1,818 |
| 79 | limit 78 to yr="2004 -Current" | 1,107 |

**Table S2: Search strategies in OVID Embase**

Embase Classic+Embase <1947 to 2024 May 01>

| **#** | **Query** | **Results from 2 May 2024** |
| --- | --- | --- |
| 1 | disabled person/ | 44,949 |
| 2 | daily life activity/ | 121,423 |
| 3 | disabled.tw,kw. | 37,506 |
| 4 | disabilit*.tw,kw. | 370,426 |
| 5 | handicap*.tw,kw. | 38,399 |
| 6 | (activit* adj1 daily living).tw,kw. | 3,216 |
| 7 | physically disabled person/ | 201 |
| 8 | physical disability/ | 27,223 |
| 9 | walking difficulty/ | 17,216 |
| 10 | (physical* adj limit*).tw,kw. | 4,879 |
| 11 | (physical* adj impair*).tw,kw. | 4,768 |
| 12 | motor limitation*.tw,kw. | 178 |
| 13 | motor impair*.tw,kw. | 14,139 |
| 14 | mobility limit*.tw,kw. | 2,438 |
| 15 | mobility impair*.tw,kw. | 1,929 |
| 16 | functional limitation*.tw,kw. | 12,519 |
| 17 | (functional* adj impair*).tw,kw. | 36,758 |
| 18 | activity limitation*.tw,kw. | 6,124 |
| 19 | participation limitation*.tw,kw. | 154 |
| 20 | (walk* adj difficult*).tw,kw. | 1,320 |
| 21 | ambulation difficult*.tw,kw. | 65 |
| 22 | exp self help device/ | 3,319 |
| 23 | exp mobility device/ | 21,821 |
| 24 | dependent ambulation.tw,kw. | 32 |
| 25 | assistive technolog*.tw,kw. | 5,024 |
| 26 | assistive device*.tw,kw. | 5,261 |
| 27 | wheel chair*.tw,kw. | 1,008 |
| 28 | wheelchair*.tw,kw. | 14,042 |
| 29 | mobility aid*.tw,kw. | 762 |
| 30 | mobility equipment.tw,kw. | 70 |
| 31 | mobility technolog*.tw,kw. | 66 |
| 32 | self help device*.tw,kw. | 248 |
| 33 | (walk* adj aid*).tw,kw. | 2,204 |
| 34 | amputee/ | 2,305 |
| 35 | amput*.tw,kw. | 78,575 |
| 36 | paraplegia/ | 32,442 |
| 37 | quadriplegia/ | 22,794 |
| 38 | paraplegi*.tw,kw. | 28,488 |
| 39 | quadr#plegi*.tw,kw. | 7,310 |
| 40 | tetraplegi*.tw,kw. | 7,598 |
| 41 | mentally disabled person/ | 1,902 |
| 42 | exp intellectual impairment/ | 672,866 |
| 43 | cognitive defect/ | 227,969 |
| 44 | (mental* adj retard*).tw,kw. | 49,009 |
| 45 | (cognitive* adj impair*).tw,kw. | 147,901 |
| 46 | cognitive disorder*.tw,kw. | 10,403 |
| 47 | cognitive dysfunction*.tw,kw. | 33,606 |
| 48 | developmental disorder/ or developmental delay/ | 59,518 |
| 49 | (development* adj disorder*).tw,kw. | 16,365 |
| 50 | developmental delay*.tw,kw. | 30,202 |
| 51 | learning disorder/ | 37,113 |
| 52 | learning disorder*.tw,kw. | 2,529 |
| 53 | academic disorder*.tw,kw. | 3 |
| 54 | hearing impaired person/ | 1,872 |
| 55 | hearing impairment/ | 88,289 |
| 56 | hearing impair*.tw,kw. | 21,531 |
| 57 | hearing loss.tw,kw. | 76,248 |
| 58 | deaf*.tw,kw. | 56,300 |
| 59 | hard of hearing.tw,kw. | 2,450 |
| 60 | visually impaired person/ | 9,423 |
| 61 | visual impairment/ or blindness/ or low vision/ | 112,614 |
| 62 | visual disorder/ | 38,168 |
| 63 | (visual* adj impair*).tw,kw. | 24,847 |
| 64 | vision impair*.tw,kw. | 4,573 |
| 65 | vision disorder*.tw,kw. | 1,049 |
| 66 | blind*.tw,kw. | 537,191 |
| 67 | vision loss.tw,kw. | 18,766 |
| 68 | low vision.tw,kw. | 5,243 |
| 69 | deafblindness/ | 279 |
| 70 | deafblind*.tw,kw. | 202 |
| 71 | deaf blind*.tw,kw. | 651 |
| 72 | sensory loss*.tw,kw. | 6,074 |
| 73 | sensory impair*.tw,kw. | 4,020 |
| 74 | 1 or 2 or 3 or 4 or 5 or 6 or 7 or 8 or 9 or 10 or 11 or 12 or 13 or 14 or 15 or 16 or 17 or 18 or 19 or 20 or 21 or 22 or 23 or 24 or 25 or 26 or 27 or 28 or 29 or 30 or 31 or 32 or 33 or 34 or 35 or 36 or 37 or 38 or 39 or 40 or 41 or 42 or 43 or 44 or 45 or 46 or 47 or 48 or 49 or 50 or 51 or 52 or 53 or 54 or 55 or 56 or 57 or 58 or 59 or 60 or 61 or 62 or 63 or 64 or 65 or 66 or 67 or 68 or 69 or 70 or 71 or 72 or 73 | 2,371,878 |
| 75 | exp hysterectomy/ | 99,536 |
| 76 | hysterectom*.tw,kw. | 74,951 |
| 77 | 75 or 76 | 110,957 |
| 78 | 74 and 77 | 4,194 |
| 79 | limit 78 to yr="2004 -Current" | 3,265 |

**Table S3: Search strategies in OVID APA PyscINFO**

APA PsycInfo <1806 to April Week 4 2024>

| **#** | **Query** | **Results from 2 May 2024** |
| --- | --- | --- |
| 1 | disabilities/ | 23,931 |
| 2 | "activities of daily living"/ | 7,127 |
| 3 | disabled.tw. | 30,796 |
| 4 | disabilit*.tw. | 146,260 |
| 5 | handicap*.tw. | 23,163 |
| 6 | (activit* adj1 daily living).tw. | 670 |
| 7 | physical disorders/ | 14,297 |
| 8 | physical mobility/ | 3,087 |
| 9 | (physical* adj limit*).tw. | 1,062 |
| 10 | (physical* adj impair*).tw. | 1,748 |
| 11 | motor limitation*.tw. | 70 |
| 12 | motor impair*.tw. | 3,977 |
| 13 | mobility limit*.tw. | 475 |
| 14 | mobility impair*.tw. | 534 |
| 15 | functional limitation*.tw. | 2,486 |
| 16 | (functional* adj impair*).tw. | 9,165 |
| 17 | activity limitation*.tw. | 1,247 |
| 18 | participation limitation*.tw. | 58 |
| 19 | (walk* adj difficult*).tw. | 126 |
| 20 | ambulation difficult*.tw. | 7 |
| 21 | assistive technology/ | 3,116 |
| 22 | mobility aids/ | 1,380 |
| 23 | dependent ambulation.tw. | 4 |
| 24 | assistive technolog*.tw. | 2,677 |
| 25 | assistive device*.tw. | 905 |
| 26 | wheel chair*.tw. | 66 |
| 27 | wheelchair*.tw. | 2,023 |
| 28 | mobility aid*.tw. | 186 |
| 29 | mobility equipment.tw. | 14 |
| 30 | mobility technolog*.tw. | 29 |
| 31 | self help device*.tw. | 30 |
| 32 | (walk* adj aid*).tw. | 172 |
| 33 | amputation/ | 1,200 |
| 34 | amput*.tw. | 2,586 |
| 35 | paraplegia/ | 714 |
| 36 | quadriplegia/ | 246 |
| 37 | paraplegi*.tw. | 1,493 |
| 38 | quadr#plegi*.tw. | 665 |
| 39 | tetraplegi*.tw. | 453 |
| 40 | intellectual development disorder/ | 43,817 |
| 41 | cognitive impairment/ | 46,294 |
| 42 | (mental* adj retard*).tw. | 42,767 |
| 43 | (cognitive* adj impair*).tw. | 52,208 |
| 44 | cognitive disorder*.tw. | 3,579 |
| 45 | cognitive dysfunction*.tw. | 9,477 |
| 46 | developmental disabilities/ | 14,176 |
| 47 | (development* adj disorder*).tw. | 8,700 |
| 48 | developmental delay*.tw. | 5,767 |
| 49 | learning disabilities/ | 22,478 |
| 50 | learning disorder*.tw. | 2,381 |
| 51 | academic disorder*.tw. | 8 |
| 52 | hearing disorders/ | 8,974 |
| 53 | hearing loss/ or deafness/ | 17,031 |
| 54 | hearing impair*.tw. | 7,227 |
| 55 | hearing loss.tw. | 10,477 |
| 56 | deaf*.tw. | 19,143 |
| 57 | hard of hearing.tw. | 2,815 |
| 58 | vision disorders/ | 8,349 |
| 59 | blindness/ or low vision/ | 5,889 |
| 60 | (visual* adj impair*).tw. | 6,192 |
| 61 | vision impair*.tw. | 763 |
| 62 | vision disorder*.tw. | 251 |
| 63 | blind*.tw. | 63,136 |
| 64 | vision loss.tw. | 1,100 |
| 65 | low vision.tw. | 1,205 |
| 66 | deaf blind/ | 353 |
| 67 | deafblind*.tw. | 228 |
| 68 | deaf blind*.tw. | 548 |
| 69 | sensory loss*.tw. | 799 |
| 70 | sensory impair*.tw. | 1,229 |
| 71 | 1 or 2 or 3 or 4 or 5 or 6 or 7 or 8 or 9 or 10 or 11 or 12 or 13 or 14 or 15 or 16 or 17 or 18 or 19 or 20 or 21 or 22 or 23 or 24 or 25 or 26 or 27 or 28 or 29 or 30 or 31 or 32 or 33 or 34 or 35 or 36 or 37 or 38 or 39 or 40 or 41 or 42 or 43 or 44 or 45 or 46 or 47 or 48 or 49 or 50 or 51 or 52 or 53 or 54 or 55 or 56 or 57 or 58 or 59 or 60 or 61 or 62 or 63 or 64 or 65 or 66 or 67 or 68 or 69 or 70 | 419,593 |
| 72 | hysterectomy/ | 499 |
| 73 | hysterectom*.tw. | 915 |
| 74 | 72 or 73 | 939 |
| 75 | 71 and 74 | 73 |
| 76 | limit 75 to yr="2004 -Current" | 56 |

**Table S4: Search strategies in EBSCO CINAHL Plus**

| **#** | **Query** | **Limiters/Expanders** | **Results** |
| --- | --- | --- | --- |
| S73 | S68 AND S71 | Limiters - Publication Date: 20040101-20241231  Search modes - Boolean/Phrase | 424 |
| S72 | S68 AND S71 | Search modes - Boolean/Phrase | 547 |
| S71 | S69 OR S70 | Search modes - Boolean/Phrase | 13,765 |
| S70 | TI hysterectom* OR AB hysterectom* | Search modes - Boolean/Phrase | 11,253 |
| S69 | (MH "Hysterectomy") | Search modes - Boolean/Phrase | 7,945 |
| S68 | S1 OR S2 OR S3 OR S4 OR S5 OR S6 OR S7 OR S8 OR S9 OR S10 OR S11 OR S12 OR S13 OR S14 OR S15 OR S16 OR S17 OR S18 OR S19 OR S20 OR S21 OR S22 OR S23 OR S24 OR S25 OR S26 OR S27 OR S28 OR S29 OR S30 OR S31 OR S32 OR S33 OR S34 OR S35 OR S36 OR S37 OR S38 OR S39 OR S40 OR S41 OR S42 OR S43 OR S44 OR S45 OR S46 OR S47 OR S48 OR S49 OR S50 OR S51 OR S52 OR S53 OR S54 OR S55 OR S56 OR S57 OR S58 OR S59 OR S60 OR S61 OR S62 OR S63 OR S64 OR S65 OR S66 OR S67 | Search modes - Boolean/Phrase | 480,674 |
| S67 | TI "sensory impair*" OR AB "sensory impair*" | Search modes - Boolean/Phrase | 1,102 |
| S66 | TI "sensory loss*" OR AB "sensory loss*" | Search modes - Boolean/Phrase | 941 |
| S65 | TI "deaf blind*" OR AB "deaf blind*" | Search modes - Boolean/Phrase | 234 |
| S64 | TI deafblind* OR AB deafblind* | Search modes - Boolean/Phrase | 221 |
| S63 | (MH "Deaf-Blind Disorders") | Search modes - Boolean/Phrase | 334 |
| S62 | TI "low vision" OR AB "low vision" | Search modes - Boolean/Phrase | 1,448 |
| S61 | TI "vision loss" OR AB "vision loss" | Search modes - Boolean/Phrase | 2,880 |
| S60 | TI blind* OR AB blind* | Search modes - Boolean/Phrase | 99,811 |
| S59 | TI "vision disorder*" OR AB "vision disorder*" | Search modes - Boolean/Phrase | 115 |
| S58 | TI "vision impair*" OR AB "vision impair*" | Search modes - Boolean/Phrase | 1,049 |
| S57 | TI "visual* impair*" OR AB "visual* impair*" | Search modes - Boolean/Phrase | 6,398 |
| S56 | (MH "Vision Disorders") OR (MH "Blindness") OR (MH "Vision, Subnormal") | Search modes - Boolean/Phrase | 18,150 |
| S55 | TI "hard of hearing" OR AB "hard of hearing" | Search modes - Boolean/Phrase | 1,681 |
| S54 | TI deaf* OR AB deaf* | Search modes - Boolean/Phrase | 10,105 |
| S53 | TI "hearing loss" OR AB "hearing loss" | Search modes - Boolean/Phrase | 18,976 |
| S52 | TI "hearing impair*" OR AB "hearing impair*" | Search modes - Boolean/Phrase | 5,338 |
| S51 | (MH "Hearing Disorders") OR (MH "Deafness") | Search modes - Boolean/Phrase | 28,549 |
| S50 | TI "academic disorder*" OR AB "academic disorder*" | Search modes - Boolean/Phrase | 0 |
| S49 | TI "learning disorder*" OR AB "learning disorder*" | Search modes - Boolean/Phrase | 491 |
| S48 | (MH "Learning Disorders") | Search modes - Boolean/Phrase | 7,555 |
| S47 | TI "developmental delay*" OR AB "developmental delay*" | Search modes - Boolean/Phrase | 4,443 |
| S46 | TI "development* disorder*" OR AB "development* disorder*" | Search modes - Boolean/Phrase | 2,737 |
| S45 | (MH "Developmental Disabilities") | Search modes - Boolean/Phrase | 11,698 |
| S44 | TI "cognitive dysfunction*" OR AB "cognitive dysfunction*" | Search modes - Boolean/Phrase | 4,744 |
| S43 | TI "cognitive disorder*" OR AB "cognitive disorder*" | Search modes - Boolean/Phrase | 1,279 |
| S42 | TI "cognitive* impair*" OR AB "cognitive* impair*" | Search modes - Boolean/Phrase | 33,797 |
| S41 | TI "mental* retard*" OR AB "mental* retard*" | Search modes - Boolean/Phrase | 3,819 |
| S40 | (MH "Cognition Disorders") OR (MH "Mild Cognitive Impairment") | Search modes - Boolean/Phrase | 36,505 |
| S39 | (MH "Intellectual Disability+") | Search modes - Boolean/Phrase | 36,782 |
| S38 | (MH "Persons with Intellectual Disabilities") | Search modes - Boolean/Phrase | 5,321 |
| S37 | TI tetraplegi* OR AB tetraplegi* | Search modes - Boolean/Phrase | 2,083 |
| S36 | TI quadr?plegi* OR AB quadr?plegi* | Search modes - Boolean/Phrase | 1,347 |
| S35 | TI paraplegi* OR AB paraplegi* | Search modes - Boolean/Phrase | 3,907 |
| S34 | (MH "Quadriplegia") | Search modes - Boolean/Phrase | 3,347 |
| S33 | (MH "Paraplegia") | Search modes - Boolean/Phrase | 2,965 |
| S32 | TI amput* OR AB amput* | Search modes - Boolean/Phrase | 15,102 |
| S31 | (MH "Amputees") | Search modes - Boolean/Phrase | 3,470 |
| S30 | TI "walking aid*" OR AB "walking aid*" | Search modes - Boolean/Phrase | 685 |
| S29 | TI "self help device*" OR AB "self help device*" | Search modes - Boolean/Phrase | 10 |
| S28 | TI "mobility technolog*" OR AB "mobility technolog*" | Search modes - Boolean/Phrase | 40 |
| S27 | TI "mobility equipment" OR AB "mobility equipment" | Search modes - Boolean/Phrase | 61 |
| S26 | TI "mobility aid*" OR AB "mobility aid*" | Search modes - Boolean/Phrase | 323 |
| S25 | TI wheelchair* OR AB wheelchair* | Search modes - Boolean/Phrase | 5,824 |
| S24 | TI "wheel chair*" OR AB "wheel chair*" | Search modes - Boolean/Phrase | 78 |
| S23 | TI "assistive device*" OR AB "assistive device*" | Search modes - Boolean/Phrase | 2,048 |
| S22 | TI "assistive technolog*" OR AB "assistive technolog*" | Search modes - Boolean/Phrase | 3,276 |
| S21 | TI "dependent ambulation" OR AB "dependent ambulation" | Search modes - Boolean/Phrase | 10 |
| S20 | (MH "Assistive Technology Devices") OR (MH "Ambulation Aids+") OR (MH "Wheelchairs+") | Search modes - Boolean/Phrase | 14,100 |
| S19 | TI "ambulation difficult*" OR AB "ambulation difficult*" | Search modes - Boolean/Phrase | 11 |
| S18 | TI ( "walking difficult*" OR "difficult* walking" ) OR AB ( "walking difficult*" OR "difficult* walking" ) | Search modes - Boolean/Phrase | 595 |
| S17 | TI "participation limitation*" OR AB "participation limitation*" | Search modes - Boolean/Phrase | 75 |
| S16 | TI "activity limitation*" OR AB "activity limitation*" | Search modes - Boolean/Phrase | 2,445 |
| S15 | TI "function* impair*" OR AB "function* impair*" | Search modes - Boolean/Phrase | 9,010 |
| S14 | TI "functional limitation*" OR AB "functional limitation*" | Search modes - Boolean/Phrase | 4,863 |
| S13 | TI "mobility impair*" OR AB "mobility impair*" | Search modes - Boolean/Phrase | 802 |
| S12 | TI "mobility limit*" OR AB "mobility limit*" | Search modes - Boolean/Phrase | 973 |
| S11 | TI "motor impair*" OR AB "motor impair*" | Search modes - Boolean/Phrase | 2,732 |
| S10 | TI "motor limitation*" OR AB "motor limitation*" | Search modes - Boolean/Phrase | 46 |
| S9 | TI ("physical* impair*") OR AB ("physical* impair*") | Search modes - Boolean/Phrase | 1,734 |
| S8 | TI ("physical* limit*") OR AB ("physical* limit*") | Search modes - Boolean/Phrase | 1,308 |
| S7 | (MH "Physical Mobility") | Search modes - Boolean/Phrase | 7,823 |
| S6 | TI ("activit* of daily living") OR AB ("activit* of daily living") | Search modes - Boolean/Phrase | 19,498 |
| S5 | TI handicap* OR AB handicap* | Search modes - Boolean/Phrase | 6,468 |
| S4 | TI disabilit* OR AB disabilit* | Search modes - Boolean/Phrase | 128,132 |
| S3 | TI disabled OR AB disabled | Search modes - Boolean/Phrase | 12,100 |
| S2 | (MH "Activities of Daily Living") | Search modes - Boolean/Phrase | 39,200 |
| S1 | (MH "Persons with Disabilities") | Search modes - Boolean/Phrase | 37,598 |

**Table S5:** Details of the Newcastle Ottawa Scale

| **Cohort Studies** | | | | | | | | | |
| --- | --- | --- | --- | --- | --- | --- | --- | --- | --- |
| **First Author Last Name, Year** | **1) Representativeness of the exposed cohort** | **2) Selection of the non exposed cohort** | **3) Ascertain-ment of exposure** | **4) Demonstration that outcome of interest was not present at start** | **5) Compar-ability of cohorts (design or analysis)** | **6) Assessment of outcome** | **7) Follow-up long enough for outcomes to occur** | **8) Adequacy of follow up of cohorts** | **Final**  **Possible Score** |
| Possible Response | Truly representative of the average in the community*  Somewhat representative of the average in the community*  Selected group  No description | Drawn from the same community as the exposed cohort*  Drawn from a different source  No description | Secure record*  Structured interview*  Self-report  No description | Yes*  No | Study controls for age*  Study controls for age and comorbidities or socioeconomic status** | Independent blind assessment*  Record linkage*  Self-report  No description | Yes*  No | Complete follow up*  Follow up rate ≥ 80%*  Follow up rate < 80%  No description | 9 |
| **Cross-sectional studies** | | | | | | | | | |
|  | **1) Representativeness of the participants** | **2) Selection of the non exposed cohort** | **3) Ascertain-ment of exposure** | **4) Demonstration that outcome of interest was not present at start** | **5) Compar-ability of cohorts (design or analysis)** | **6) Assessment of outcome** | **7) Follow-up long enough for outcomes to occur** | **8) Adequacy of follow up of cohorts** | **Final**  **Possible Score** |
| Possible Response | Truly representative of the average in the community*  Somewhat representative of the average in the community*  Selected group  No description | N/A | Secure record*  Structured interview*  Self-report  No description | N/A | Study controls for age*  Study controls for age and comorbidities or socioeconomic status** | Independent blind assessment*  Record linkage*  Self-report  No description | N/A | N/A | 5 |

**Table S6**: Studies that were excluded during full-text screening and reasons for exclusion

| **Study** | **Reason for exclusion** |
| --- | --- |
| Amir et al., 2022^1^ | Wrong population – only included individuals that had hysterectomy |
| Fakas et al., 2022^2^ | Wrong exposure – did not focus on disability specifically (i.e., looked at social vulnerability in general) |
| Gorina et al., 2024^3^ | Not peer-reviewed article – government report |
| Grimstad et al., 2020^4^ | Not peer-reviewed article – only abstract available |
| Ho et al., 2018^5^ | Wrong exposure – did not focus on disability (i.e., exclusively focused on patients with uterine fibroids) |
| Lin et al., 2012^6^ | Wrong or no comparison group – only included individuals with disabilities |
| Marquez-Gonzalez et al., 2021^7^ | Wrong population – only included individuals that had hysterectomy and age criteria unclear |
| Scime et al., 2023^8^ | Wrong population – only included individuals that had hysterectomy |
| Valdez-Martinez et al., 2024^9^ | Not in English/French – only available in Spanish |

**References**

1. Amir N, Mitra M, Leung K, Moore Simas TA. Complications following hysterectomy in women with intellectual and developmental disabilities. *Disabil Health J*. 2022;15(1):101213. doi:10.1016/j.dhjo.2021.101213

2. Fakas S, Lu AMR, Shahani D, Blitz MJ, Rodriguez-Ayala G. Social Vulnerability Index and Surgical Management of Abnormal Uterine Bleeding in Reproductive-age Women. *J Minim Invasive Gynecol*. 2022;29(9):1104-1109. doi:10.1016/j.jmig.2022.06.003

3. Gorina Y, Elgaddal N, Weeks JD, Pingali C, Valenzuela C. *Hysterectomy Among Women Age 18 and Older: United States, 2021​*. National Center for Health Statistics (U.S.); 2024. doi:10.15620/cdc:145592

4. Grimstad F, Gaddis M, Strickland J. 117. Utilizing National Data to Evaluate Persistence of Hysterectomies Performed in Youth with Developmental Delay: are we Improving? *J Pediatr Adolesc Gynecol*. 2020;33(2):232. doi:10.1016/j.jpag.2020.01.049

5. Ho YL, Li CS, Liu CC, Lin CC, Hung CJ, Kao CH. Disability benefits as an incentive for hysterectomy: Uterine fibroid patients in Taiwan. *Women Health*. 2018;58(8):866-883. doi:10.1080/03630242.2017.1358793

6. Lin LP, Hsieh M, Chen SF, Wu CL, Hsu SW, Lin JD. Factors related to hysterectomy in women with physical and mobility disabilities. *Res Dev Disabil*. 2012;33(4):990-995. doi:10.1016/j.ridd.2012.01.002

7. Márquez-González H, Valdez-Martínez E, Bedolla M. Clinical, Epidemiologic and Ethical Aspects of Hysterectomy in Young Females With Intellectual Disability: A Multi-Centre Study of Public Hospitals in Mexico City. *Front Public Health*. 2021;9:746399. doi:10.3389/fpubh.2021.746399

8. Scime NV, Brown HK, Metcalfe A, Simpson AN, Brennand EA. Bilateral salpingo-oophorectomy at the time of benign hysterectomy among females with disabilities: a population-based cross-sectional study. *Am J Obstet Gynecol*. 2023;229(6):658.e1-658.e17. doi:10.1016/j.ajog.2023.08.001

9. Valdez-Martínez E, Márquez-González H, Bedolla M. Histerectomía no terapéutica en jóvenes mexicanas con discapacidad intelectual: una realidad problematizada. *Gac Médica México*. 2024;160(2):179-186. doi:10.24875/GMM.23000460
